# Near-Infrared Confocal Reflectance Scanning Laser Ophthalmoscopy (SLO) and Short-Wavelength Autofluorescence Imaging in Cystic Diabetic Macular Edema

**DOI:** 10.1155/2022/6831396

**Published:** 2022-05-30

**Authors:** Fariba Ghassemi, Fatemeh Bazvand, Houshang Faghihi, Ramak Roohipourmoallai, Maryam Masoumi, Sepide Jamali, Masoumeh Mohebbi, Siamak Sabour

**Affiliations:** ^1^Eye Research Center, Farabi Eye Hospital, Tehran University of Medical Sciences, Tehran, Iran; ^2^The Retina and Vitreous Surgery Service, Farabi Eye Hospital, Tehran University of Medical Sciences, Tehran, Iran; ^3^Departmant of Ophthalmology, Morsani College of Medicine, University of South, Tampta, FL, USA; ^4^Safety Promotion and Injury Prevention Research Center, Shahid Beheshti University of Medical Sciences, Tehran, Iran; ^5^Department of Clinical Epidemiology, Shahid Beheshti University of Medical Sciences, Tehran, Iran

## Abstract

**Objective:**

To characterize results of short-wavelength autofluorescent (SW-AF) and near-infrared confocal reflectance scanning laser ophthalmoscopy (NIR-cR SLO) imaging in cystic diabetic macular edema (DME).

**Design:**

Cross-sectional study. Participants: 104 eyes from 52 naïve treatment patients with DME and mild to moderate nonproliferative diabetic retinopathy (NPDR) Methods: complete ocular examination, best-corrected visual acuity (BCVA), and imaging were performed.

**Results:**

In NIR-cR SLO, small/medium and large-sized cysts presented with decreased and increased reflectance, respectively. In SW-AF, increased and decreased autofluorescence, corresponding to medium-/large- and small-sized cysts were noted. Mainly, the lower NIR reflectance was associated with petaloid edema pattern in SW-AF (*P*=0.011), BCVA (logMAR) (*P*=0.056), subretinal fluid (*P*=0.035), and the involved layers of retina by cysts (*P* < 0.001) in Pearson chi-square test. Fair agreement of 0.31 (*P* < 0.001) between NIR-cR SLO and late FA leakage was found by the weighted kappa test. In regression analysis, NIR-cR SLO abnormality is highly correlated with outer and inner nuclear layers location of the cystic changes.

**Conclusions:**

The size of cysts and involved layers affect presenting features of SW-AF and NIR reflectance.

## 1. Introduction

Diabetic macular edema (DME) is a main cause of visual loss in adult age, characterized by increased macular thickness [1, 2]. The common and standard diagnostic technique in detection of DME is fundoscopy by slit-lamp biomicroscopy [3]. Optical coherence tomography (OCT) is an accurate method in detecting retinal thickness even in early stage of DME [4, 5]. Infrared (IR, 870 nm), by its higher wavelength causes enhanced viewing of the retinal and subretinal structures [6].

Short wave autofluorescence (SW-AF, 488 nm) has been used mostly for structural evaluation of age-related macular degeneration, macular dystrophies, cystoid macular edema of different origins, and DME [7–16]. SW-AF is a simple technique by a reported sensitivity and specificity of 81% and 69%, respectively, in detection of cystoid edema [7, 8]. Recently, near-infrared autofluorescence (NIR-AF) has been introduced to measure the AF of the melanin in the RPE and the choroid [6, 17]. Hypofluorescence of the NIR-AF images is relative in DME, compared with the definite absence of fluorescence signals in geographic atrophy and retinitis pigmentosa [18].

NIR was found to be superior to standard color fundus photography in screening for neovascular AMD [19, 20]. NIR make it possible to detect leakage from active choroidal neovascularization (CNV) and different types of neovascular AMD [21].

Autofluorescence (AF) is thought to derive from lipofuscin in retinal pigment epithelial (RPE) cells, reflecting some aspect of RPE function and integrity in contrast to NIR signal from the melanin as the main fluorophore in the RPE and the choroid [7, 13, 21–24]. According to the literature, the distribution or intensity of the fluorescence signals on NIR reflectance images in DME and the relationship to the characteristics on SD-OCT images is poorly understood. For the first time, herein, we report the qualitative findings of NIR-cR SLO image versus SW-AF and other image modalities and evaluate the association of the findings with macular thickening, visual acuity, and quantitative and qualitative parameters of OCT findings in cystic DME.

## 2. Methods

This retrospective study was carried out in Farabi Eye Hospital Tehran University of Medical Sciences from 2014 to 2015 after ethics committee approval. The study adhered to the tenets of the Declaration of Helsinki, and the informed consent was achieved from all patients. The consequent patients who were diagnosed as center and noncenter involving DME with mild to moderate nonproliferative diabetic retinopathy on fundoscopy DME and the availability of same day taken routine SD-OCT, fluorescein angiography (FA), NIR-cR SLO, SW-AF, and IR images were included in the study. The patients with any other chorioretinal diseases, dense cataract, proliferative diabetic retinopathy, and/or any recent 6-months history of ocular surgery were excluded.

After comprehensive ophthalmologic examinations including measurement of best-corrected VA taken with Snellen chart, slit-lamp biomicroscopy, color fundus photography, SW-AF (inciting 488 nm and emitted light >500 nm), and IR images with 870 nm (superluminescence diode) of the macula were obtained using a scanning laser ophthalmoscope, using the 30° and 55° field of view (Heidelberg Retinal Angiograph 2; Heidelberg, Germany). The optical and technical principles of the HRA have been described in detail [17, 21, 25–27]. For SW-AF, five to nine images at 512 × 512 pixels resolution were taken consequently and averaged using the HRA mean algorithm supplied. SW-AF examination was performed before FA when both examinations were planned at the same day. The IR (870 nm) was evaluated for hyperreflective, hyporeflective, and mix (hyper and hypo) lesion at the cystic spaces in OCT.

OCT scanning and NIR-cR SLO imaging (815 nm filter) were performed using SD-OCT (Spectralis OCT; Heidelberg Engineering), and the OCT parameters were evaluated quantitatively and qualitatively. Raster scans were taken to get the mean central subfield (CSF) thickness and OCT map constructed, as described previously [27, 28]. The cysts were classified according to 200-*µ*m scale on the SD-OCT images. As the largest diameter of cyst was classified into 3 categories including small (<100 *µ*m cysts or those confined to only a single layer), medium (≥100 *µ*m and <200 *µ*m or those in at most 2 layers), and large cyst (≥200 *µ*m or those in at least in 3 layers) in OCT [28]. Then, in different cuts of OCT, the image characteristics of different sizes of cysts were evaluated on SLO IR and AF.

Angiographic characteristics were evaluated for both early and late phases FA at the cystic spaces on SD-OCT.

For fundus photography, a conventional fundus camera (Topcon TRC50LX, Topcon, Tokyo, Japan) with a halogen lamp exciter with a 580 nm band-pass filter (bandwidth 500–610 nm) and a 695 nm barrier filter (bandwidth 675–715 nm) was obtained as a single image.

Grading of the images was performed independently by two masked readers (FG and FB), and in case of disagreement, a senior retinal specialist (HF) acted as arbitrator. Normal decreased foveal autofluorescence due to luteal pigment was considered as normal.

### 2.1. Statistical Analysis

The data were reported in mean ± standard deviation (SD) and were analyzed by SPSS version 19 (SPSS Inc., Chicago, IL, USA). The Pearson correlation was evaluated between BCVA and central foveal thickness and OCT volume as well as FA edema patterns with SW-AF and NIR-cR SLO. Agreement between different parameters was measured by the weighted kappa test. The significance of the relationship between FA parameters and SW-AF, NIR-cR SLO, and other imaging modalities characteristics were evaluated by means of Chi-square Pearson test. Furthermore, we performed multiple logistic regression analysis to control the effect of confounders in evaluating the efficacy of NIR-cR SLO and SW-AF abnormalities in cystic areas. *P* < 0.05 was considered significant.

## 3. Results

One hundred and four eyes from 52 patients (male: 25) with the mean age of 57.5 ± 10.5 years old (range: 30–76 years) were evaluated. The mean BCVA was 0.4 ± 0.3 in LogMAR (Table 1). The mean of central foveal thickness (CFT) and volume in OCT were 387.7 ± 129.5 *µ*m and 10.7 ± 1.7 mm3, respectively. A significant correlation was found among BCVA (LogMAR), CFT (*r* = 0.347, *P*=0.001), and OCT volume (*r* = 0.344, *P*=0.001) (Pearson's correlation). The cystic edema was observed in 99 (96.1%) eyes on OCT images as the gold standard imaging for cystic changes. The subfoveal cyst was found in 48 eyes (46.6%). Outer nuclear (ONL) and inner nuclear layer (INL) and interim outer plexiform layer (OPL) were the most commonly involved layers followed by ONL alone.

OCT volume showed a direct correlation with OCT thickness (*r* = 0.72, *P*=0.001) as expected and pattern of leakage on FA (*r* = 0.44, *P*=0.001). OCT large cyst presence showed a stronger correlation with the hyperfluorescence in the early phase of FA (*r* = 0.43, *P*=0.001) than late phase hyperfluorescence (*r* = 0.34, *P*=0.01). Lower than 0.4 correlation coefficients were computed for the remaining relationships.

### 3.1. The Characteristics of Cysts Sizes in Different Imaging Modalities

**Figure 1:**
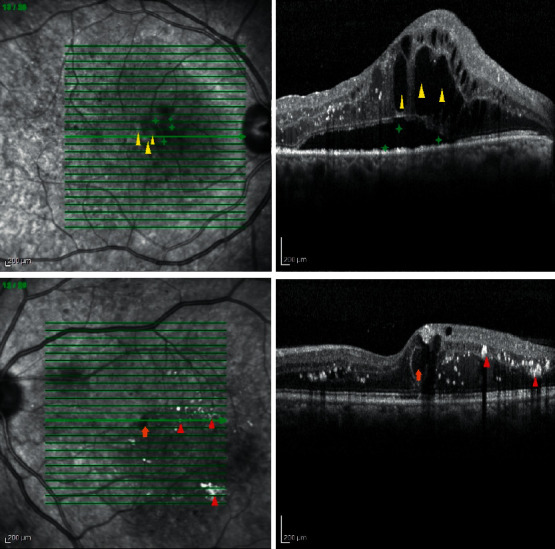
Near-infrared reflectance taken with OCT SLO image with related OCT image (left and right images, respectively) shows hyperreflectivity at the site of large cysts (yellow arrowhead) and hyporeflectivity at the site of subretinal fluid (green plus) in top row images. NIR also shows hyporeflectivity at the site of medium cyst (orange arrow) and hyperreflectivity at the site of exudates (red arrowhead) at bottom row images.

**Figure 2:**
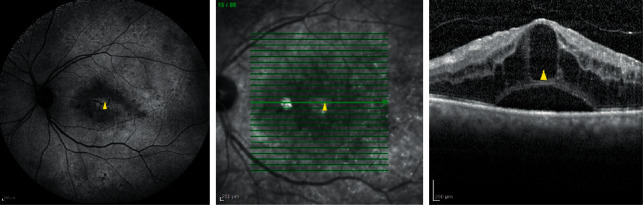
Autofluorescence (left image) and near-infrared reflectance (middle image) taken with OCT SLO image with related OCT image (right image) show hyperautofluorescence and hyperreflectivity at the site of large cyst (yellow arrowhead) and hypoautofluorescence and hyporeflectivity around it concordance with subretinal fluid.

According to the size of the cysts in SD-OCT, different image modalities showed diverse appearances (Tables 2 and 3, and Figures 1 and 2). No large cyst was seen alone in studied eyes. By evaluating the cystic areas in different images, twenty-nine (33.0%) eyes had a normal AF pattern, 39 (44.3%) eyes had single-spot, multiple-spot, and petaloid increased AF, and 20 (22.7%) eyes showed decreased AF at the site of cystic changes according to OCT images (Table 2). NIR-cR SLO showed hyporeflective in 49 (66.2%) eyes, isoreflective in 17 (23%) eyes, and hyperreflective signals in 8 (10.8%) eyes. On FA, 38 (36.8%) eyes had focal leakage and 66 (63.5%) eyes showed diffuse leakage at cystic areas. Intergrader agreement was almost perfect (*k* = 0.80; 95% CI, 0.74–0.86).

## 4. Association between SW-AF Petaloid Pattern and Other Image Modalities Findings

SW petaloid hyperfluorescent pattern was associated with early (Pearson chi-square, *P*=0.018) and late FA leakages pattern (Pearson chi-square, *P*=0.009), and no relations was found with BCVA, volume, and CFT.

### 4.1. Association between NIR Confocal Hyporeflectance Pattern and Other Image Modalities Findings

NIR-cR, mainly hyporeflectance, was associated with the petaloid SW-AF pattern (Pearson chi-square, *P*=0.011), overall AF edema pattern (Pearson chi-square, *P*=0.001), BCVA (logMAR) (Pearson chi-square, *P*=0.056), subretinal fluid (Pearson chi-square, *P*=0.035), OCT layers involved by the cysts (Pearson chi-square, *P*=0.001), and OCT type of the cysts (Pearson chi-square, *P*=0.001), both early and late fluorescence in FA (Pearson chi-square, *P*=0.001), and IR reflectance (Pearson chi-square, *P*=0.001). The only agreement that was found is a 0.31 (*P*=0.001) fair agreement between NIR-cR SLO and late FA leakage, by weighted kappa.

### 4.2. Association between NIR-cR SLO and SW-AF Images Abnormality and Morphologic Parameters

Unadjusted analysis showed strong association of NIR-cR SLO with the location and type of cysts. We tried to evaluate more of this association after adjusting for some confounding factors (age and sex). A logistic regression analysis was carried out to evaluate the association between NIR-cR parameters and the layers involved by cysts on OCT. The analysis showed that after adjusting abnormal NIR (mostly hyporeflective condition), there is 6.1 times probability of ONL involvement alone (OR = 6.13, 95% CI = 1.02–36.67, *P*=0.017). In addition, this probability for simultaneous ONL and INL involvement is 4.4 times (OR = 4.47, 95% CI = 1.31–15.22, *P*=0.047). In a similar regression analysis for SW-AF, no significant result was obtained in our data set. It means that AF image characteristics are independent of cysts location and sizes in the retina, as expected.

## 5. Discussion

Considering OCT as the gold standard in the diagnosis of cystic edema, NIR-cR SLO is comparable to SW-AF in detecting cystic changes. To the best of our knowledge, this is the first study evaluating NIR-cR SLO and SW-AF characteristics with the location of the cystic changes in DME, in the performed PubMed and Scopus English literature review. Although, OCT is gold standard in diagnosis of diabetic edema and cystic changes, in this study, we would like to evaluate the characteristics of DME based on the size of cysts categorized based on OCT. SLO IR and AF images may be useful in determine of response to treatment based on the image characteristics or may be helpful as guide for macular laser photocoagulations that requires future studies. On the other hand, use of SLO IR and AF images may help to find biomarkers in prognosis, response to treatment, and as a probable guide in macular laser photocoagulation in future studies. For example, in a study by Murakami et al., they found that the granular pattern (hyper- and hyporeflective lesions) in SW-AF and NIR-AF was associated with thicker retina, ELM disruption, and hyperreflective foci in outer retinal layers [29]. They found the granular pattern was a sign of photoreceptor damage in diabetic macular edema and visual acuity reduction [12]. In another study, it was shown that the mosaic pattern in the NIR-AF and cystoids change in NIR-AF was associated with worse VA [30].

In contrast to SW-AF excitation, NIR-cR SLO abnormality is highly correlated with cystic changes in ONL and INL in DME. In the study of Bessho et al., 488 nm AF showed a petaloid autofluorescence pattern in macula in all 14 studied eyes (100%) [12]. In contrast, by 580 nm AF, just one case (6.6%) showed a petaloid autofluorescence pattern [12]. The discrepancies observed between our study and other studies [8, 12] could be due to wavelength differences for AF like that observed by Bessho et al.

We found that in all size cystic edemas considering OCT findings, SW-AF was often isofluorescent (55.4%). This amount for increased autofluorescence in the study of Vujosevic et al. was 77–89% [10]. This difference could be due to the special technique for images acquisition. For amplifying the autofluorescence signal of the final image, they aligned 15 acquired images and the mean one was calculated by image analysis software. In our study, 8 consequent images were averaged for getting the final image. SW-AF is thought to visualize the distribution of lipofuscin in the RPE [7, 13, 14, 30, 31]. The two hypotheses were proposed for explanation of increased AF in cystic edema [10]. The first one was the lipofuscin as indicator of oxidative stress and probable origin of fluorescence in SW-AF accumulated in microglia rather than RPE. The other is the recognized activation of microglia in diabetes [10, 32]. Our analysis did not support this hypothesis. The second theory was proposed according to most prevalent layers (OPL and INL) involved in both cystic edema and as principle locations of luteal pigment that normally blocks the fluorescence especially in fovea [8, 10]. The mechanical displacement of retinal layers induced by cystic change could attenuate the luteal pigment in these layers and resultant increased SW-AF fluorescence [10]. Our study showed no association between the cysts location and SW-AF characteristics. We believe that the drying effect of RPE on retina in DME could explain the accumulated lipofuscin as a stress pigment in this layer. This hyperfunctioning is more prominent in cystic DME.

Yoshitake evaluated the association of hypofluorescence in the macula on the NIR autofluorescence with SD-OCT images in DME [27]. They concluded that NIR hypofluorescent features were delineated in the areas corresponding to serous retinal detachment (SRD) and cystoid spaces on SD-OCT images [27]. They speculated that the observed hypofluorescence could be due to partial depletion of RPE melanin or associated fluorophores in the RPE or the choroid, accumulated nonabsorbable extravasated blood components from the retinal vasculature through the damaged RPE in diabetic patients [27]. In our study, there was a weak positive correlation between NIR reflectance and subretinal fluid (*r* = 0.23, *p*=0.035). In NIR illumination, blood components, including hemoglobin and oxygenated hemoglobin as well as water, are the most important potential absorbing pigments in the normal fundus; thus, large, perfused vessels appear dark in vivo [6].

In our study, the observed NIR hyporeflectivity was associated with normal IR (870 nm) at the cystic areas of the retina. Because of the normality of IR, the hyporeflective areas could not be due to RPE or choroidal melanin pigment content as Bartsch et al. stated [27]. This could result from higher hemoglobin and water content of both intermediate and deep capillary plexus, in accordance with more correlation of hyporeflectivity in NIR-cR SLO with small- or medium-sized cyst located in INL and ONL. Lack of hyporeflectivity of NIR reflectance at the large sized cysts could be due to 400 *μ*m wide capillary-free region, centered around the fovea, as usual site of large cysts. Presence of NIR-absorbing chromophores in these small- and medium-sized cysts is another hypothesis.

We could rely on NIR-cR SLO on detecting the edema in outer layers of retina. Although, we are unable to consider the importance of NIR hyporeflectance in ONL and INL involving cysts, it may serve to generate working hypotheses for future in more extensive studies. It could lead us to study the possible evaluation of retinal layers by different wavelengths of lasers. The layers of human fundus contain a variety of absorbing, reflecting, and scattering materials which differ significantly across individuals [33, 34]. If we can correlate histologic and spectrophotometric analysis of fundus changes with the multiwavelength imaging pattern, this noninvasive in vivo analysis of fundus changes could potentially help to discriminate between subretinal [24] and intraretinal reflective materials.

Our study has several limitations attributable mainly to its retrospective design and to the limited included eyes and nonrepeatability of the imaging in patients. This analysis did not allow us to understand the correlation of NIR-cR SLO and outer retinal changes. Future studies will investigate these subjects.

## 6. Conclusions

To the best of our knowledge, NIR-cR SLO characteristics of cystic DME have not been reported in detail before. SW-AF and NIR-cR SLO imaging provide valuable information regarding the cysts in macular area. The cystic portions of macula were observed as hyporeflective areas in medium and small cysts by NIR-cR SLO imaging. The hyporeflective NIR-cR SLO could address to retinal outer and inner nuclear layer location of the cystic changes in DME.

## Figures and Tables

**Table 1:** General characteristics of patients with CSME.

Age (mean ± SD)	57.5 (±10.5)
Sex (male), *n* (%)	50 (48.1)
BCVA (LogMAR, SD)	0.4 (±0.3)
OCT central foveal thickness, *μ*m (mean ± SD)	387.7 (±129.5)
OCT macular volume, mm^3^ (mean ± SD)	10.7 (±1.7)
Overall SW-AF pattern, *n* (%)	
Petaloid	49 (59%)
Nonpetaloid (single-spot, multiple-spot, and normal)	34 (41%)
Overall FA pattern, *n* (%)	
Petaloid	32 (30.8)
Nonpetaloid	72 (69.2)
FA leakage extend, *n* (%)	
Focal	38 (36.8)
Diffuse	66 (63.5)
OCT edema patterns, *n* (%)	
Center involved cystoid	48 (46.2)
Noncentered cystoid	56 (53.8)
Subfoveal neuroretinal detachment	30 (28.9)
Pure noncystoid (spongelike)	11 (10.6)
Cystoid (overall)	89 (89.4)

SW-AF: short-wave autofluorescence.

**Table 2:** Description of cystic diabetic macular edema patterns by different image modalities characteristics of patients with CSME.

	Frequencies, *N* (%)
Fundus photo	
Cystic	37 (35.6)
Absent foveal reflex	84 (80.8)
OCT findings	
ONL, OPL, INL	20 (19.2)
ONL, INL	42 (40.4)
ONL	28 (28.8)
IPL, INL	1 (1)
None	11 (10.6)
NIR reflectance (815)	
ISO	34 (32.7)
Hypo	61 (58.7)
Hyper	9 (8.7)
IR (870) reflectance	
ISO	58 (55.8)
Hypo	19 (18.3)
Hyper	24 (23.1)
SW-AF	
ISO	29 (33.0)
Hypo	20 (22.7)
Hyper	39 (44.3)
Early FA fluorescence	
ISO	40 (38.5)
Hypo	50 (48.1)
Hyper	14 (13.5)
Late FA fluorescence	
ISO	23 (22.1)
Hypo	9 (8.7)
Hyper	72 (69.2)

INL: inner nuclear layer; IPL: inner plexiform layer; IR: infrared; FA: fluorescein angiography; NIR: near-infrared confocal reflectance; OCT: optical coherence tomography; ONL: outer nuclear layer; OPL: outer plexiform layer; SRF: subretinal fluid; SW-AF: short-wave autofluorescence.

**Table 3:** Association of the type of cystic changes in SD-OCT with different presentation patterns in common imaging modalities.

Type of imaging			Type of cystic edema					
			Large + medium *N* (%)	Medium + small *N* (%)	Medium *N* (%)	Small *N* (%)	Mix *N* (%)	*P*value chi-square Pearson
Fundus Photogram	Absent		10 (12.1)	28 (34.2)	9 (11.0)	28 (34.2)	7 (8.5)	<0.001
	Present		4 (36.4)	2 (18.1)	1 (9.1)	4 (36.4)	0	

SW-AF	ISO		3 (10.7)	14 (48.8)	1 (3.4)	10 (34.8)	0	0.07
	Hyper		7 (20.0)	13 (37.1)	6 (17.4)	7 (20.0)	2 (5.5)	
	Hypo		1 (6.3)	1 (6.3)	1 (6.3)	10 (62.4)	3 (18.7)	

NIR (815)	ISO		1 (2.9)	3 (8.8)	2 (5.9)	17 (50.0)	0	<0.001
	Hyper		5 (55.6)	3 (33.3)	1 (11.1)	0	0	
	Hypo		8 (13.1)	24 (39.3)	7 (11.5)	15 (24.6)	7 (11.5)	

IR (870)	ISO		6 (11.5)	14 (26.9)	3 (5.8)	18 (34.6)	2 (3.8)	0.16
	Hypo		3 (15.8)	6 (31.6)	5 (26.3)	4 (21.1)	1 (5.3)	
	Hyper		4 (16.7)	6 (25.0)	2 (8.3)	8 (33.3)	2 (8.3)	
	Mix		0	2 (66.7)	0	0	1 (33.3)	

FA	Early	ISO	0	7 (23.3)	2 (20)	20 (62.5)	0	<0.001
		Hypo	10 (20)	19 (38.0)	7 (14.0)	10 (20.0)	4 (8.0)	
		Hyper	4 (28.6)	4 (28.6)	1 (7.1)	2 (14.3)	3 (21.4)	
	Late	ISO	0	3 (20.0)	0	12 (80.0)	0	<0.001
		Hypo	0	2 (22.2)	2 (22.2)	3 (33.4)	2 0(22.2)	
		Hyper	14 (20.3)	25 (43.4)	8 (11.5)	17 (24.6)	5 (7.2)	

## Data Availability

The datasets during and/or analysed during the current study are available from the corresponding author on reasonable request

## Abbreviations:

SW-AF: short-wavelength autofluorescent
NIR-cR SLO: near-infrared confocal reflectance scanning laser ophthalmoscopy
DME: Diabetic macular edema
NPDR: Nonproliferative diabetic retinopathy
BCVA: Best-corrected visual acuity
OCT: Optical coherence tomography
IR: Infrared
CNV: Choroidal neovascularization
RPE: Retinal pigment epithelial
FA: Fluorescein angiography
CSF: Central subfield
CFT: Central foveal thickness
ONL: Outer nuclear
INL: Inner nuclear layer
OPL: Interim outer plexiform layer.
